# Effects of maggot antimicrobial peptides on growth performance, immune function, and cecal flora of yellow-feathered broilers

**DOI:** 10.3389/fvets.2023.1156964

**Published:** 2023-07-25

**Authors:** Shengjie Gao, Quancheng Zhang, Caixia Liu, Hong Shen, Jungang Wang

**Affiliations:** ^1^College of Animal Science and Technology, Shihezi University, Shihezi, China; ^2^College of Agriculture, Shihezi University, Shihezi, China

**Keywords:** yellow-feathered broilers, maggot antimicrobial peptide, growth performance, immune index, cecal microbiota

## Abstract

**Introduction:**

This study investigated the effects of maggot antimicrobial peptides on growth performance, blood parameters, immune organ index, and cecum microbial diversity in yellow broilers.

**Methods:**

The addition of 100–300 mg/kg maggots antimicrobial peptides to the corn-soybean meal basal diet was evaluated. Two hundred and forty one-day-old yellow-feathered broilers were randomly divided into four groups (60 chickens in each group): basal diet group (BC group), basal diet group + 100, 200, 300 mg/kg maggots antimicrobial peptides (MDAL group, MDAM group, and MDAH group).

**Results:**

The result showed that the average daily feed intake (**ADFI**) of the BC group, MDAM group, and MDAH group was higher than that of the MDAL group (*P* > 0.05), the average daily gain of MDAM group and MDAH group was significantly higher than that of BC group and MDAL group (*P* < 0.05), but the feed-weight ratio (**F/G**) was significantly lower than that of BC group (*P* < 0.05). The total protein (**TP**) content in the MDAM group and MDAH group was significantly higher than that in the BC group (*P* < 0.05), and the albumin (**ALB**) content in the MDAH group was higher than that in the BC group (*P* > 0.05). The contents of IgA and IgG in the MDAH group were significantly higher than those in the BC group (*P* < 0.05). In contrast, the content of alanine aminotransferase (**ALT**) in the MDAH group was significantly lower than that in the BC group (*P* < 0.05). The thymus and spleen indexes of the MDAH group were significantly higher than those of the BC group (*P* < 0.05). 16S rDNA sequencing results showed that *Bacteroidota* and *Bacteroides* were the dominant phylum and genus of cecal microorganisms at the phylum and genus levels, respectively. Cecum microorganisms are mainly involved in biological processes such as energy production and conversion, amino acid transport and metabolism, and carbohydrate transport and metabolism.

**Discussion:**

It was concluded that adding different doses of maggot antimicrobial peptide to the basal diet could improve yellow-feathered broilers' growth and immune performance and change the cecum flora. The appropriate dose of antimicrobial peptide addition was 300 mg/kg.

## Introduction

With the successful treatment of animal diseases by antibiotics, the emergence and rapid spread of drug-resistant bacteria pose a significant threat to human health (1). Antibiotic resistance and residues have become an increasingly severe problem (2). Therefore, there is an urgent need to develop new antibiotics, and antimicrobial peptides are an alternative (3). Antimicrobial peptides are essential for innate immunity (4). Due to their broad-spectrum activities and different mechanisms from antibiotics, antimicrobial peptides reduce the risk of bacterial resistance and are considered an ideal choice for replacing antibiotics (5).

In recent years, antimicrobial peptides have been widely used as new antimicrobial agents in pig and poultry production (6). Much literature has shown that antimicrobial peptides can improve the growth performance of pigs and poultry (7–9). Antimicrobial peptide CAP benefits the growth performance, diarrhea rate, apparent digestibility, and fecal flora of weaned piglets, which can be a potential substitute for antibiotic growth promoters (10). Antimicrobial peptides A3 and P5 promoted broiler growth performance with similar effects to the antibiotic-treated group (11). In addition, adding porcine antimicrobial peptides in drinking water or feed can also promote the growth performance of broilers (12). Antimicrobial peptides are the effector molecules of innate and adaptive immunity, which regulate pro-inflammatory and anti-inflammatory responses and chemotaxis and can directly affect adaptive immunity (13). It was found that the addition of compound antimicrobial peptides composed of pig defensins and housefly antimicrobial peptides in diets could increase the number of T cells in weaned piglets, enhance the proliferation of peripheral blood T cells, and reduce the percentage of spleen cell apoptosis (14). Antimicrobial peptides also have beneficial effects on intestinal microflora in broilers. Previous studies have shown that antimicrobial peptides could significantly reduce the number of *Clostridium perfringens* in the cecum of broilers, reduce the impact of harmful bacteria on the growth of broilers and reduce the risk of infection (15).

Insects are nature's most significant biological groups, with rich antimicrobial peptide resources (16). As an essential resource insect, housefly larvae are rarely infected by pathogens in harsh environments such as decaying substances, feces, and garbage containing many bacteria (17). It is mainly because maggots contain active substances such as antimicrobial peptides, lysozyme, and lectins that kill and inhibit bacteria, fungi, and viruses (18). Our previous work has shown that maggots' antimicrobial peptides can inhibit *Escherichia coli, Staphylococcus, Salmonella*, and other pathogens (3, 13, 19) and enough to treat chickens infected with *E. coli* and *Salmonella* (20, 21). Further studies found that maggot antimicrobial peptides increased immunity and enhanced resistance to pathogenic bacteria by increasing red blood cells in the blood and regulating the expression of immune factors in the intestinal mucosa of dysentery chickens (22–24). Recent studies have shown that combined fly maggot antimicrobial peptides, and astragalus polysaccharides can promote yellow-feathered broilers' growth performance, slaughter performance, and immune organ index (25). However, it is not clear how maggots antimicrobial peptides enhance chicken immunity by regulating the intestinal flora and changing the diversity and richness of the flora.

Here, we study the growth performance, blood parameters, and immune organ index of yellow-feathered broilers in each experimental group were determined by adding different doses of fly maggot antimicrobial peptides to the diet of yellow feathered broilers. Based on the analysis of cecal flora by 16S rDNA high-throughput sequencing, the effects of different doses of fly maggot antimicrobial peptides on the growth performance and immune function of yellow-feathered broilers were evaluated, which provided a theoretical basis for the application of fly maggot antimicrobial peptides as an alternative to antibiotics in livestock and poultry breeding.

## Materials and methods

The study was approved by the Animal Experimentation Ethics Committee of the School of Animal Science and Technology, Shihezi University. The code of ethical inspection was A2021-14. All chickens were kept experimentally and euthanized per the committee's guidelines. During the test, all efforts were made to minimize the suffering of the animals.

### The animals and dietary composition

Molecular Biology Laboratory, College of Animal Science and Technology, Shihezi University, prepared antimicrobial peptides from maggots. Yellow feather broilers, purchased from Three-three Hatchery, Shihezi, China. The essential diet was the corn-soybean meal, formulated according to the national standard of the People's Republic of China's “Laying Hens and Broilers Compound Feed” (GB-T5916-2020). The diet composition and nutritional level are shown in Table 1.

**Table 1:** Composition and nutritional level of essential experimental diet (air-dried basis, %).

**Item**	**1–21 days old**	**22–42 days old**	**43–63 days old**
**Raw material**			
Corn	60.85	62.65	68.30
Soybean meal	28.00	25.30	19.65
Cottonseed protein	2.00	2.00	2.00
Corn protein powder	2.00	2.00	2.00
Peanut oil	2.70	3.80	4.00
Stone powder	1.10	1.10	1.00
CaHPO_4_	1.60	1.40	1.30
NaCl	0.30	0.30	0.30
Na_2_CO_3_	0.15	0.15	0.15
Lys	0.10	0.10	0.10
Met	0.10	0.10	0.10
Thr	0.10	0.10	0.10
Premix ^a^	1.00	1.00	1.00
Total	100.00	100.00	100.00
**Nutritional level** ^b^			
Metabolizable energy (MG/kg)	12.47	12.87	13.14
Crude protein	20.39	19.36	17.40
Ca	0.97	0.91	0.82
Total phosphorus	0.75	0.70	0.66
Available phosphorus	0.45	0.41	0.38
Lys	1.08	1.01	0.87
Met	0.43	0.42	0.39
Thr	0.79	0.76	0.71

^*a*^The premix provides per kilogram of feed: VA 1500 IU, VD 3200 IU, VE 10 mg, VK 0.5 mg, VB 1.8 mg, VB 3.5 mg, VB 0.01 mg, niacin 15 mg, calcium pantothenate 10 mg, biotin 0.15 mg, folic acid 0.55 mg, choline chloride 1,000 mg, Fe 80 mg, Cu 8 mg, Mn 80 mg, Zn 40 mg, I 0.35 mg, Se 0.3 mg.

^*b*^Nutrient levels were all calculated values.

### Experiment design and feeding management

The average weight of 240 healthy male yellow-feather broilers at one day old was 48.03 g. There was no statistical difference in weight. In this study conducted with a one-factor, utterly randomized design, 240 chickens were randomly divided into four treatments, each with six replicates and 10 broilers per replicate. All the broilers were in routine feeding management with free intake and drinking water. The experimental period was 63 d. The grouping is indicated in Table 2.

**Table 2:** The grouping of experiments.

**Group**	**Treatment**	**Number**
BC group	basal diet	60
MDAL group	basal diet + 100 mg/kg antimicrobial peptide	60
MDAM group	basal diet + 200 mg/kg antimicrobial peptide	60
MDAH group	basal diet + 300 mg/kg antimicrobial peptide	60

### Determination of growth performance and blood parameter analysis

The initial body weight of each replicate was measured at one day of age, and the body weight of each replicate was weighed at 10:00 in the morning of 63 days of age, fasting for 12 h before weighing. The feed amount and residual feed amount of each replicate were accurately recorded every day; the average daily feed intake (ADFI), average daily gain (ADG), and the feed-weight ratio of the yellow feather broiler were calculated (26).

At 63 days old, two wing veins of experimental chickens were randomly selected from each replicate for blood collection. The blood samples were centrifuged at 2,500 r/min for 20 min, and the serum was separated (27). The serum was loaded in a 2 mL EP tube and stored in a refrigerator at −20°C for further use. This study used a chicken ELISA kit to determine the contents of serum immunoglobulin G (IgG) and immunoglobulin A (IgA). The kit was purchased from Shanghai Jining Biotechnology Co., Ltd. Serum biochemical indexes were analyzed by the rapid kit method. Serum total protein (TP) was determined by the BCA method; serum albumin (ALB) was determined by the bromocresol green method; serum aspartate aminotransferase (AST) and alanine aminotransferase (ALT) were determined by Wright's method, and the kit was purchased from Nanjing Jiancheng Bioengineering Institute.

### Collection and detection of cecum contents

On the 63rd day, three yellow feather broilers were randomly selected from the experimental and control groups, with 12 broilers total. The selected yellow feather broiler cervical dislocation euthanasia after opening the abdominal cavity, separating abdominal organs to remove the cecum segment, cutting out the contents, liquid nitrogen froze, and −80°C preservation (28).

16S rDNA sequencing was entrusted to Shanghai Meiji Biomedical Technology Co., Ltd. for detection. The collected cecum content samples were sent to Shanghai Meiji Biomedical Technology Co., Ltd. for detection. The sequencing process was as follows: the total DNA in the cecum content samples was extracted, and the extracted genomic DNA was detected by 1% agarose gel electrophoresis. Using universal primers 338F and 806R for PCR amplification, the PCR products of the same sample were mixed and detected by 2% agarose gel electrophoresis. The PCR products were recovered by AxyPrep DNA gel recovery kit (AXYGEN company) and eluted by Tris_HCl. Two percentage agarose electrophoresis, and then quantitative fluorescence detection. Miseq amplicon sequencing of 16S V3-V4 region; the sequencing data were spliced, quality controlled, and de-jointed to obtain the optimized sequence. The OTU clustering was performed based on the optimized sequence to obtain the OTU abundance table for subsequent bioinformatics analysis.

The 16S rDNA sequencing results were clustered using Uparse 7.0.1090 (http://www.drive5.com/uparse/). Use the R version 3.3.1 tool to produce grade abundance curves, dilution curves, and Wayne diagrams. Alpha diversity was analyzed by Mothur 1.30.2 software (https://www.mothur.org/wiki/Download_mothur). Beta diversity distance was calculated using Qiime 1.9.1 (http://qiime.org/install/index.html) software to generate water abundance tables for each taxon. The COG function prediction of the 16S sequence is realized by PICRUSt 1.1.0 (http://picrust.github.io/picrust/~software).

### Statistical analysis

Microsoft Office Word 2016 software was used to sort out the experimental data. SPSS20.0 statistical software was used to perform a one-way analysis of variance for the experimental data. The least significant difference (LSD) method was used for multiple comparisons among the experimental groups. The results were expressed as mean ± standard deviation.

## Results

### Growth performance of broilers

The results showed that the average daily feed intake of the MDAL group was the lowest at 87.66 g, and that of the BC group, MDAM group, and MDAH group was higher than that of the MDAL group, with no significant difference (*P* > 0.05, Table 3). The average daily gain of the MDAM group and MDAH group was significantly higher than that of the BC group and MDAL group (*P* < 0.05), and the feed-weight ratio was significantly lower than that of the BC group (*P* < 0.05, Table 3).

**Table 3:** Effect of the antibacterial peptide from maggots on growth performance of yellow feather broilers.

	**BC group**	**MDAL group**	**MDAM group**	**MDAH group**
ADFI (g/d)	89.07 ± 0.70	87.66 ± 2.38	88.77 ± 1.58	89.78 ± 0.60
ADG (g/d)	35.42 ± 0.73^b^	35.82 ± 0.50^b^	36.97 ± 0.27^a^	37.07 ± 0.37^a^
F/G	2.52 ± 0.07^a^	2.45 ± 0.03^ab^	2.40 ± 0.06^b^	2.42 ± 0.03^b^

ADFI, average daily feed intake; ADG, average daily gain; F/G, feed/gain. Note: Results are expressed as mean ± standard deviation, and peer data with different lowercase letters on the shoulder indicate significant differences (P < 0.05), while the same letter or no letter on the shoulder indicate non-significant differences (P > 0.05).

### Plasma index and immune organ index of broilers

The total protein content in the MDAM group and MDAH group was significantly higher than that in the BC group (*P* < 0.05), and the albumin content in the MDAH group was higher than that in the BC group (*P* > 0.05, Table 4). The IgG content in the MDAM group and MDAH group was significantly higher than that in the BC group (*P* < 0.05), and the IgA content in the MDAH group was significantly higher than that in the BC group (*P* < 0.05, Table 4). The content of ALT in the MDAM group and MDAH group was significantly lower than in the BC group (*P* < 0.05, Table 4). Compared with the BC group, the content of AST in the MDAL group, MDAM group, and MDAH group decreased by 1.62, 2.09, and 1.76%, respectively (*P* > 0.05, Table 4).

**Table 4:** Effect of antimicrobial peptides from fly maggot on serum indexes of yellow feather broilers.

	**BC group**	**MDAL group**	**MDAM group**	**MDAH group**
TP (mg/ml)	32.57 ± 0.82^b^	33.44 ± 0.53^ab^	33.83 ± 0.31^a^	33.80 ± 0.74^a^
ALB (mg/ml)	13.57 ± 0.41	13.44 ± 0.12	13.20 ± 0.71	13.78 ± 0.38
IgG (mg/ml)	2.43 ± 0.05^b^	2.50 ± 0.04^ab^	2.54 ± 0.03^a^	2.57 ± 0.06^a^
IgA (μg/ml)	266.19 ± 3.52^b^	270.23 ± 7.26^ab^	267.54 ± 5.12^ab^	275.87 ± 3.23^a^
ALT (U/L)	8.22 ± 0.75^a^	7.37 ± 0.48^ab^	6.68 ± 0.87^b^	6.74 ± 0.80^b^
AST (U/L)	20.98 ± 1.23	20.64 ± 2.13	20.54 ± 2.77	20.61 ± 1.41

TP, total protein; ALB, albumin; IgG, immunoglobulin G; IgA, immunoglobulin A; ALT, alanine aminotransferase; AST, aminotransferase. Note: Results are expressed as mean ± standard deviation, and peer data with different lowercase letters on the shoulder indicate significant differences (P < 0.05), while the same letter or no letter on the shoulder indicate non-significant differences (P > 0.05).

The thymus index of the MDAH group was 14.55% higher than that of the BC group (*P* < 0.05), MDAL group and MDAM group were 5.09 and 2.90% higher than those in the BC group, respectively (*P* > 0.05, Table 5). The spleen index of the MDAM and MDAH groups was significantly higher than that of the BC group (*P* < 0.05, Table 5). There was no significant difference between the MDAL group and the other three experimental groups (*P* > 0.05, Table 5). There was no significant difference in the bursal of the Fabricius index among the experimental groups (*P* > 0.05, Table 5).

**Table 5:** Effect of maggot antibacterial peptide on immune organ index of yellow feather broilers (g/kg).

	**BC group**	**MDAL group**	**MDAM group**	**MDAH group**
Thymus	2.75 ± 0.21^b^	2.89 ± 0.24^ab^	2.83 ± 0.21^ab^	3.15 ± 0.11^a^
Spleen	1.73 ± 0.15^b^	1.88 ± 0.09^ab^	1.96 ± 0.08^a^	1.95 ± 0.11^a^
Bursa of Fabricius	0.74 ± 0.12	0.71 ± 0.22	0.73 ± 0.14	0.76 ± 0.04

Note: Results are expressed as mean ± standard deviation, and peer data with different lowercase letters on the shoulder indicate significant differences (P < 0.05), while the same letter or no letter on the shoulder indicate non-significant differences (P > 0.05).

### Graded abundance curve, dilution curve, and OTUs analysis of cecal microbial community

**Figure 1:**
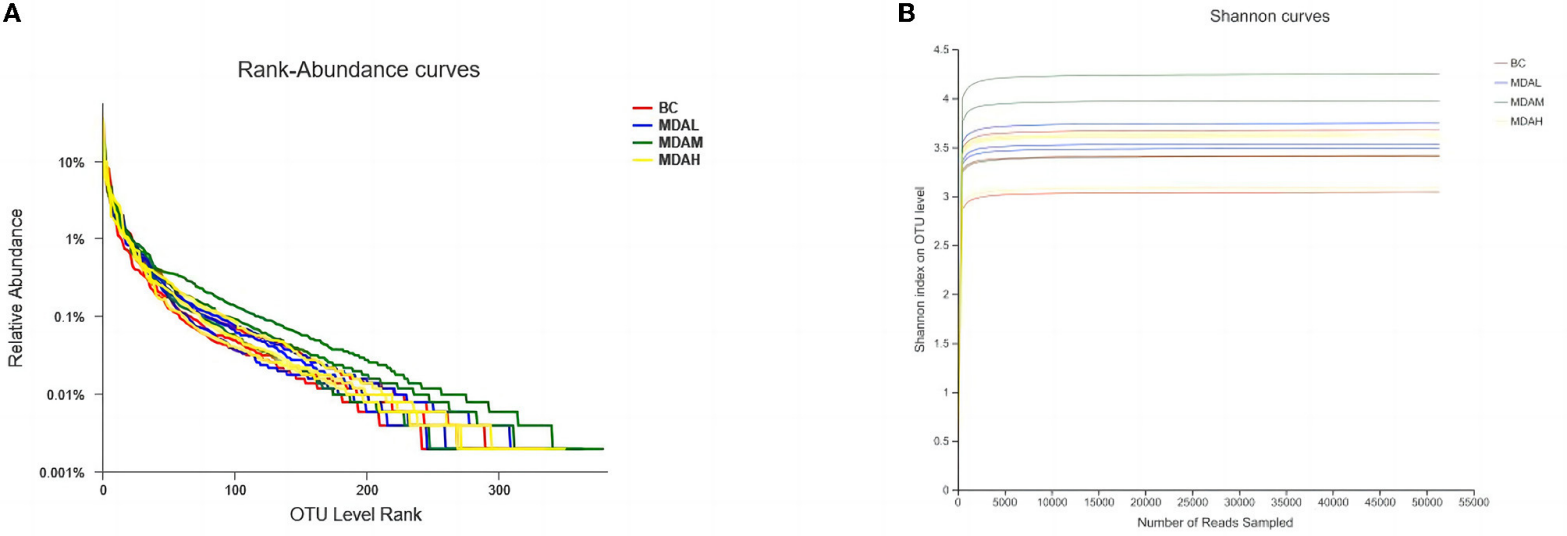
**(A)** Graded abundance cure and **(B)** dilution curve of cecal microbial community.

**Figure 2:**
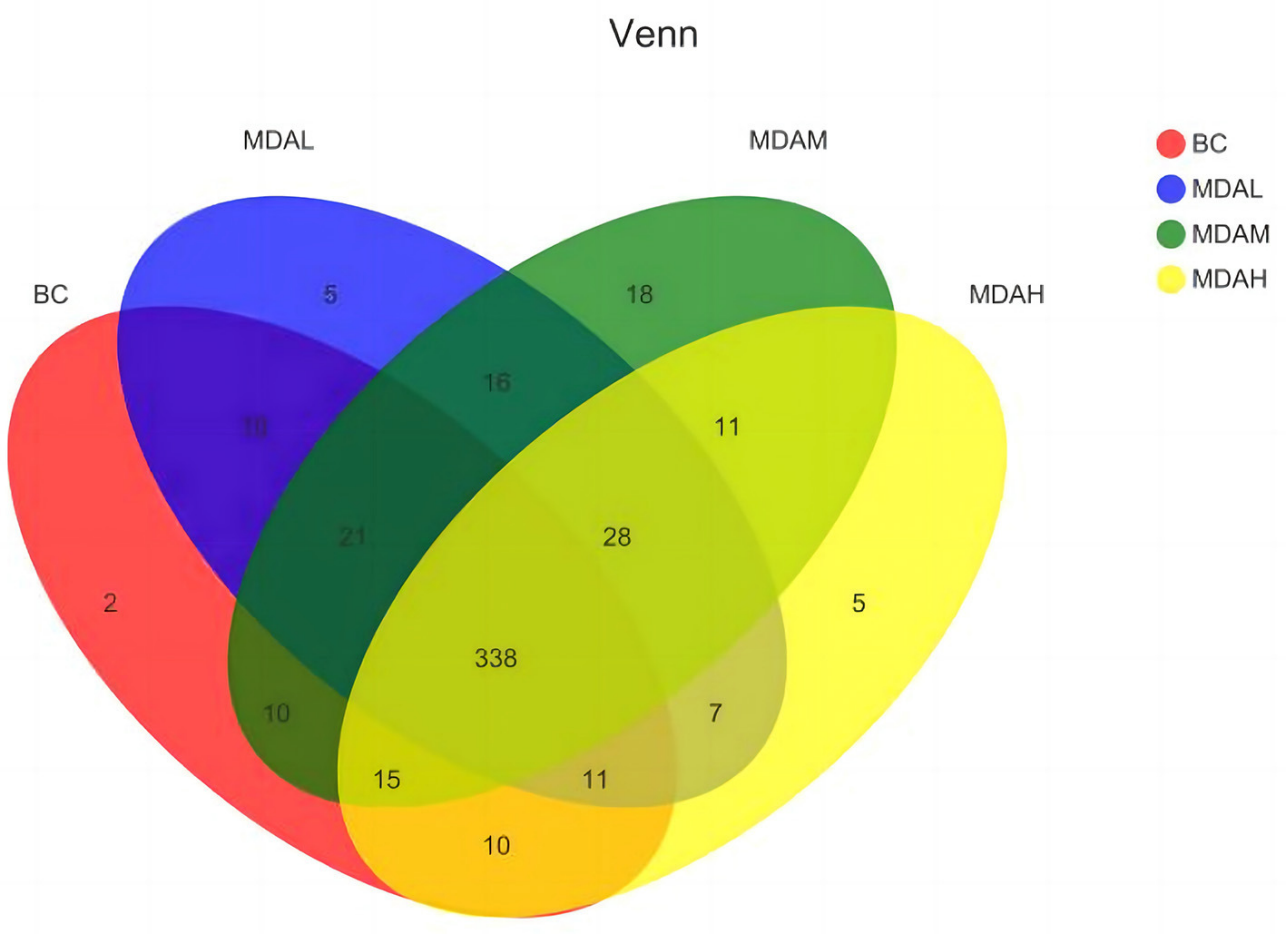
Venn diagram of cecum intestinal flora in yellow feather broilers under different treatments.

The cecum content level abundance curve of each test group became flat with the increased extraction sequencing strips (Figure 1). It indicates that the number of intestinal flora did not increase with the increase of base sequences, and the sequencing amount was sufficient to reflect the species diversity of the samples. The dilution curve tends to be flat, indicating that sequencing data is large enough to reflect the vast majority of microbial diversity information in samples (Figure 2).

There were 338 microbial OTUs in the cecal feces of yellow-feather broilers in the BC, MDAL, MDAM, and MDAH groups, accounting for 66.67% of the total 507. 417 OTUs were obtained in the BC group, with two unique ones; the number of OTUs in the MDAL group was 436, with 5 unique; in the MDAM group, 457 OTUs were obtained, with 18 unique OTUs; the number of OTUs in MDAH group was 425, unique to 5. There were 10 OTUs in the BC group and MDAL group, 10 OTUs in the BC group and MDAM group, and 10 OTUs in the BC group and MDAH group (Figure 2).

### Alpha diversity analysis and beta diversity analysis of cecal microorganisms

**Figure 3:**
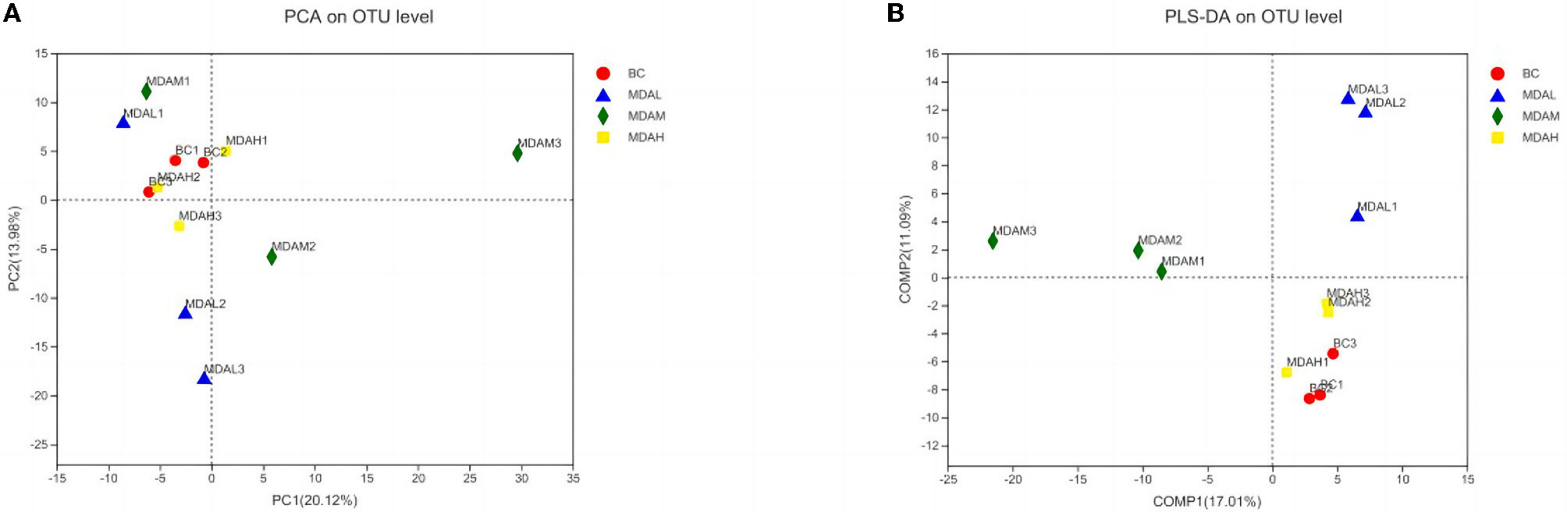
**(A)** Principal component analysis and **(B)** partial least squares discriminant analysis of cecal microflora in yellow-feather broilers with different treatments.

There was no significant difference in Shannon and Simpson index among the experimental groups (*P* > 0.05, Table 6). The Ace and Chao1 indexes of the MDAL group, MDAM group, and MDAH group were higher than those of the BC group (*P* > 0.05, Table 6). The principal component analysis showed that the microbial community composition of BC and MDAH groups was similar. However, the microbial community composition of the BC group was significantly different from that of the MDAL group and MDAM group (Figure 3A). Partial least squares discriminant analysis showed that it was easy to distinguish between groups, and three samples in the group were clustered together (Figure 3B).

**Table 6:** Determination results of microbial alpha diversity in cecum of yellow feather broilers with different treatments.

	**BC group**	**MDAL group**	**MDAM group**	**MDAH group**
Shannon	3.38 ± 0.32	3.59 ± 0.14	3.88 ± 0.43	3.44 ± 0.31
Simpson	0.09 ± 0.04	0.07 ± 0.01	0.05 ± 0.03	0.09 ± 0.04
Ace	350.98 ± 33.26	358.85 ± 48.66	384.23 ± 35.60	383.63 ± 19.98
Chao1	352.92 ± 42.38	362.14 ± 49.46	390.00 ± 19.10	390.43 ± 23.20
Good coverage	0.999	0.999	0.999	0.999

### Species relative abundance of cecal microbes at phylum and genus levels

**Figure 4:**
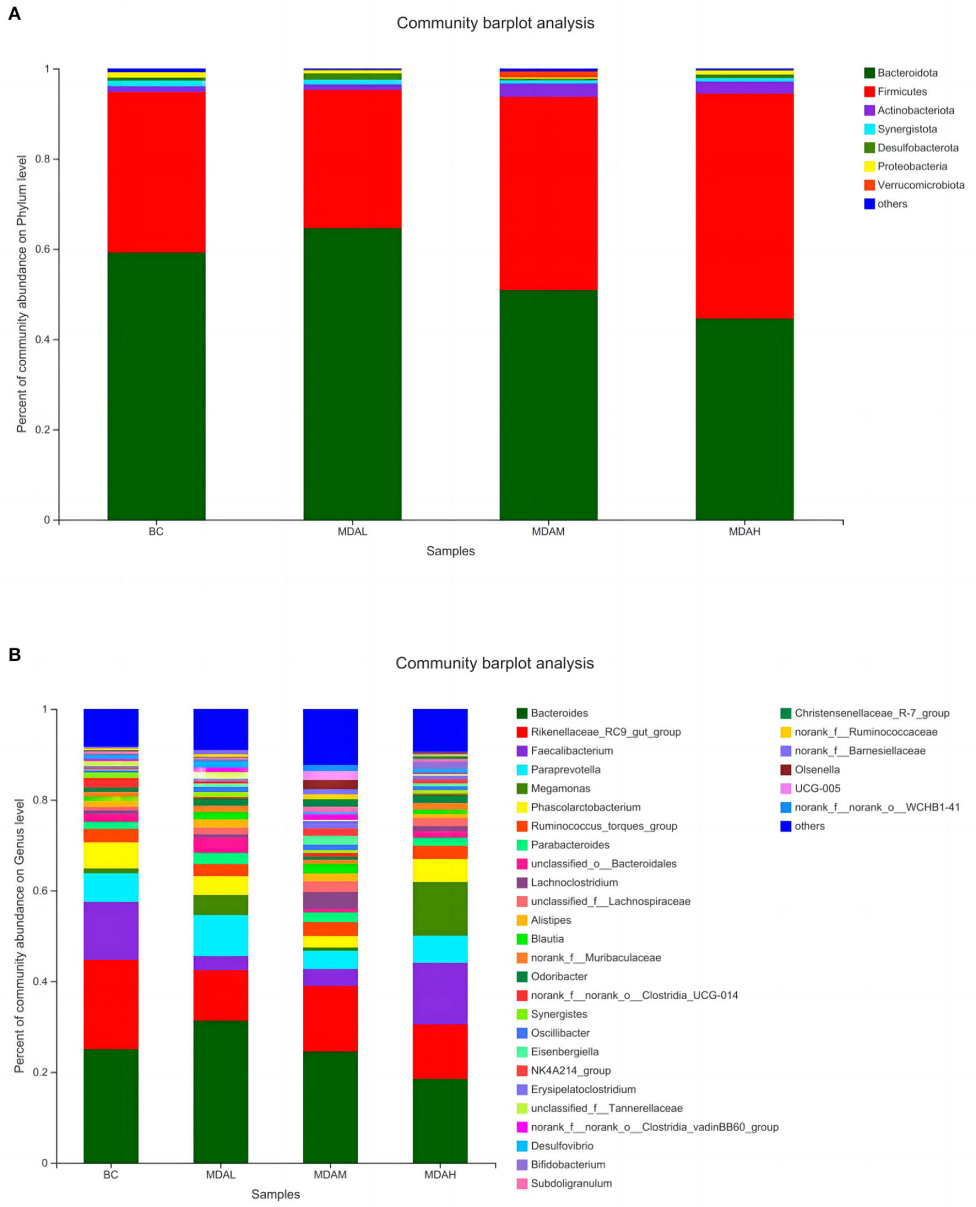
Relative abundance of microbial **(A)** phylum level species and **(B)** genus level species in cecum of yellow feather broilers under different treatments.

*Bacteroidota, Firmicutes, Actinobacteria, Synergistota, Desulfobacterota, Proteobacteria*, and *Verrucomi-crobiota* were the dominant microorganisms in the cecum of yellow-feathered broilers under different treatments (Figure 4A). *Bacteroides, Rikenellaceae_RC9_gut_group, Faecalibacterium, Paraprewohalla, Megoamonas, Phascolarctobacterium, Ruminococcus*_torques_group, *Parabacteroides, unclassified_o _Bacteroidales*, and *Lachnoclostridium* were the top 10 dominant bacteria in cecal microorganisms of yellow feathered broilers in each experimental group (Figure 4B).

The relative abundance of *Bacteroidota* in the MDAL group was significantly higher than that in the MDAH group (*P* < 0.05), and the relative abundance of Firmicutes was significantly lower than that in the MDAH group (*P* < 0.05, Table 7). The relative abundance of Firmicutes in the MDAH group was the highest, increasing by 40.11, 62.74, and 16.51%, respectively, compared with BC, MDAL, and MDAM groups (Table 7). The relative abundance of *Actinobacteriota* in the MDAL group was lower than that in the BC group, MDAM group, and MDAH group (*P* > 0.05, Table 7). The relative abundances of *Synergistota* and *Proteobacteria* in the MDAL group, MDAM group, and MDAH group were lower than those in the BC group (*P* > 0.05, Table 7). The relative abundance of Desulfobacterota in the MDAL group was significantly higher than that in the MDAM group (*P* < 0.05), and Verrucomi-crobiota in the MDAM group was higher than that in the BC group, MDAL group, and MDAH group (*P* > 0.05, Table 7).

**Table 7:** Determination results of microbial phylum levels in the cecum of yellow feather broilers with different treatments.

	**Relative abundance/%**			

	**BC group**	**MDAL group**	**MDAM group**	**MDAH group**
*Bacteroidota*	59.13^ab^	64.56^a^	50.85^ab^	44.53^b^
*Firmicutes*	35.6^ab^	30.65^b^	42.81^ab^	49.88^a^
*Actinobacteriota*	1.34	1.27	3.04	2.73
*Synergistota*	1.21	1.03	0.63	0.77
*Desulfobacterota*	0.7^ab^	1.43^a^	0.45^b^	0.79^ab^
*Proteobacteria*	1.11	0.67	0.26	0.86
*Verrucomicrobiota*	0.13	0.03	1.34	0.07

Note: Results are expressed as mean ± standard deviation, and peer data with different lowercase letters on the shoulder indicate significant differences (P < 0.05), while the same letter or no letter on the shoulder indicate non-significant differences (P > 0.05).

The dominant genera in the BC group and MDAH group were *Bacteroides, Rikenellaceae_RC9_gut_group*, and *Faecalibacterium*, the dominant genera of MDAL and MDAM groups were *Bacteroides, Rikenellaceae_RC9_gut_group* and *Paraprewohalla* (Table 8). The relative abundance of *Bacteroides* in the MDAL group was significantly higher than that in the BC group, MDAM group, and MDAH group, and that in the BC group and MDAM group was significantly higher than that in the MDAH group (*P* < 0.05, Table 8). The relative abundance of *Phascolarctobacterium* in the MDAL group, MDAM group, and MDAH group was lower than that in the BC group (*P* > 0.05), *unclassified_o_Bacteroidales* in MDAL group was significantly higher than that in the MDAM group (*P* > 0.05, Table 8).

**Table 8:** Determination results of microbial genera in the cecum of yellow feather with different treatments.

	**Relative abundance/%**			

	**BC group**	**MDAL group**	**MDAM group**	**MDAH group**
*Bacteroides*	25.02^b^	31.22^a^	24.5^b^	18.48^c^
*Rikenellaceae_RC9_gut_group*	19.67	11.12	14.46	11.99
*Faecalibacterium*	12.81	3.12	3.68	13.67
*Paraprewohalla*	6.31	9.05	4.03	5.94
*Megoamonas*	1.09	4.39	0.76	11.92
*Phascolarctobacterium*	5.73	4.17	2.51	5.01
*Ruminococcus_torques_group*	2.92	2.69	3.08	2.89
*Parabacteroides*	1.58	2.46	2.12	1.78
*unclassified_o_Bacteroidales*	1.68^ab^	3.37^a^	0.71^b^	1.38^ab^
*Lachnoclostridium*	0.83	0.73	3.76	1.83

Note: Results are expressed as mean ± standard deviation, and peer data with different lowercase letters on the shoulder indicate significant differences (P < 0.05), while the same letter or no letter on the shoulder indicate non-significant differences (P > 0.05).

### Prediction of cecal microbial function

**Figure 5:**
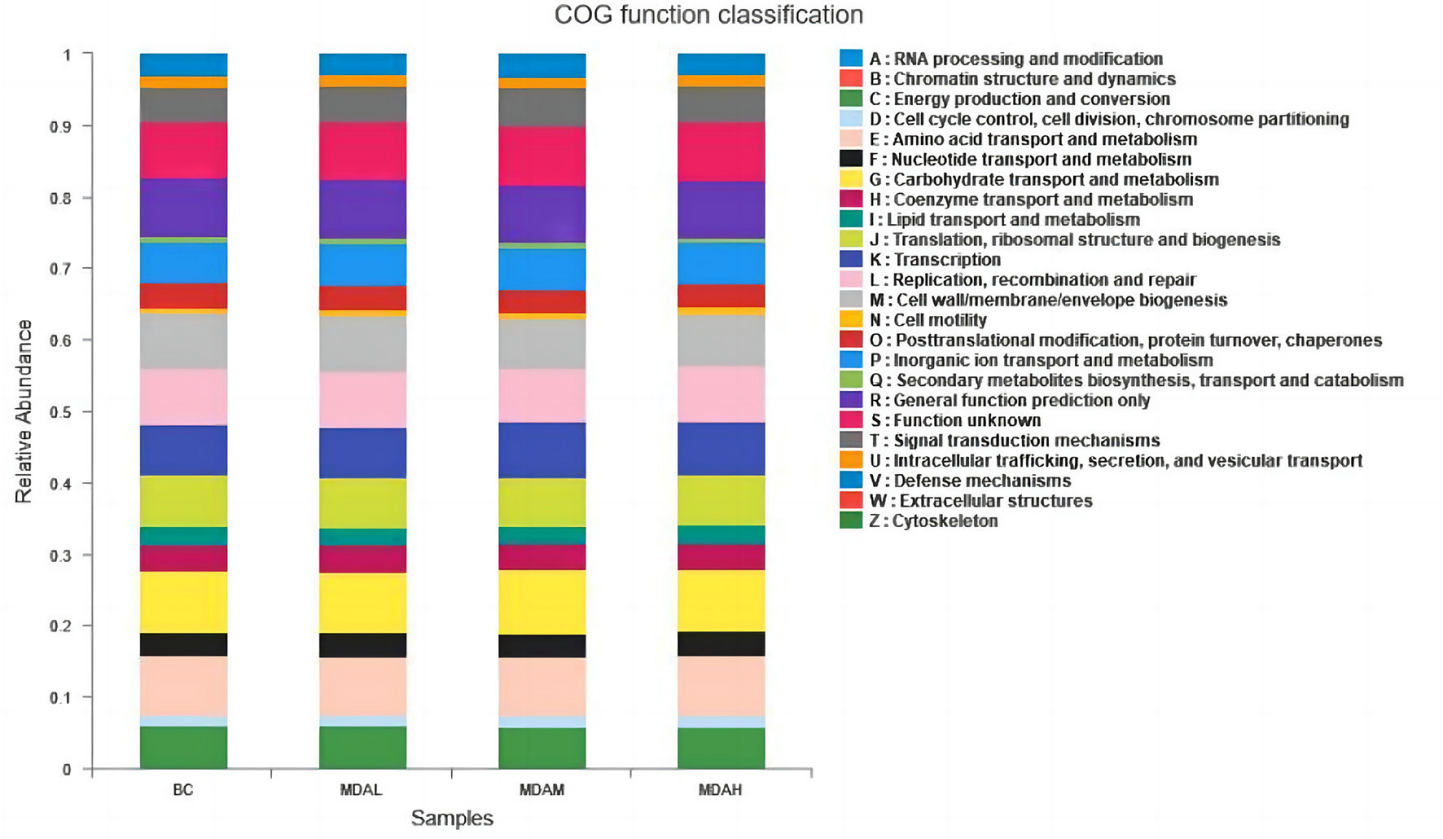
COG function classification of cecum intestinal flora in yellow feather broilers under different treatments.

Cecal microorganisms of yellow-feathered broilers is mainly involved in energy production and transformation, amino acid transport and metabolism, carbohydrate transport and metabolism, inorganic ion transport and metabolism, coenzyme transport and metabolism, lipid transport and metabolism, and other biological processes (Figure 5).

## Discussion

### Effect of maggot antimicrobial peptides on growth performance of broilers

Antimicrobial peptides can improve the growth performance of broilers (29). Studies have shown that adding antimicrobial peptide P5 to diets can significantly increase the daily gain of broilers and improve their production performance (30). In addition, it found that the addition of 100 mg/kg Pratt antimicrobial peptide and 100 mg/kg Full-tidal antimicrobial peptide in broiler diets can reduce ADFI, improve feed conversion rate and survival rate, to improve the growth performance of broilers (31). Another study showed that the addition of 20 and 30 mg/L porcine antimicrobial peptides in drinking water or 150 and 200 mg/kg porcine antimicrobial peptides in feed could improve the growth performance of broilers and the intestinal absorption of nutrients (12). This study found that MDAM and MDAH groups could significantly increase the daily gain and reduce the feed-weight ratio. It indicated that adding an antibacterial peptide of maggots in a yellow feather broiler diet could improve the growth performance of broilers.

### Effects of maggot antimicrobial peptide on serum indexes of broilers

Previous studies have shown that adding antimicrobial peptides to feed can improve animal serum indicators (32, 33). It found that adding 100 mg/kg antimicrobial peptide to the Luhua chicken diet could significantly increase the serum total protein content at 42 and 70 days of age and improve the albumin content, consistent with our study (34). Furthermore, adding 100 mg/kg antimicrobial peptide could increase the levels of IgG and IgM in broilers at 35 days of age (32). Moreover, it also found that SGAMP increased IgA levels in broiler serum (35). In this study, we found that adding an antibacterial peptide of maggots to the diet could increase the IgG and IgA content in yellow broiler's serum.

Transglutaminase mainly exists in the liver and plays a vital role in the intermediate metabolism of glucose and amino acids (33). Glutamic transaminase mainly exists in myocardial cells, the liver, and other tissues, and the activity of the two transaminases in serum is low (36). Serum glutamic-pyruvic transaminase and glutamic-oxaloacetic transaminase show vigorous activity only when liver and myocardial cells are inflammatory or damaged (37). Therefore, serum alanine aminotransferase and aspartate aminotransferase are sensitive indicators of whether hepatocytes and cardiomyocytes have inflammation and injury (38). It found that adding 100 and 200 mg/kg antimicrobial peptides to the diet of laying hens can significantly reduce the content of glutamic-oxaloacetic transaminase in serum. However, there was no significant difference in the content of glutamic-pyruvic transaminase (39). The results showed that adding 0.5% antimicrobial peptide to the diet could significantly reduce the content of alanine aminotransferase and aspartate aminotransferase in the serum of AA broilers at 42 days of age (39). This is different from the results of our study, which may be due to the different types of antimicrobial peptides and additive amounts of test materials, which need to be further verified by future experiments.

### Effects of maggot antimicrobial peptides on immune organ index of broilers

In general, the increase in the immune organ index represents good organ development. That is, the body has a solid immune function (40). It showed that the thymus and bursa of the Fabricius index reached the maximum at 18 d, and the spleen index reached the maximum at 35 d (31). Furthermore, the study found that the addition of 100–200 mg/kg Sublancin antimicrobial peptides in diets could significantly improve the liver and spleen indexes of laying hens, which played an essential role in enhancing the immune function of laying hens (41). Moreover, adding 200 mg/kg antimicrobial peptide can significantly improve the spleen index of 21-day-old Aibayijia broilers (42). Moreover, adding 200 mg/kg antimicrobial peptide to the diet could increase the thymus, spleen, and bursal index of 42-day-old broilers (43). Adding 200 and 300 mg/kg of cecropin antimicrobial peptides to the diet could significantly improve the immune organ index of 817 broilers at 21 and 42 days of age (44). In this study, we found that the thymus index and spleen index of yellow-feather broilers were increased by adding different doses of the antibacterial peptide of maggots, which was consistent with the results reported in the literature, indicating that the antibacterial peptide of maggots had a positive effect on the development of immune organs.

### Effects of maggot antimicrobial peptides on cecal microflora in broilers

Intestinal micro-ecosystem is an essential factor affecting animals' digestion and absorption ability. The balance of intestinal flora is helpful in improving the digestion and absorption ability of animals and maintaining their health of animals (45). Microbiota also plays an essential role in regulating immunity (46). There are complex microbial communities in the intestine of chickens. The diversity of intestinal flora may directly affect the physiological function of intestinal microorganisms (47). Antimicrobial peptides as a candidate substitute for antibiotics, used in livestock and poultry breeding, may have a beneficial impact on animal intestinal health. This study found that each experimental group's cecal microbial abundance curve of yellow feather broilers was smooth. It indicated that the cecal microbial evenness was high, and there was no difference in the smoothness of the dilution curve between the experimental groups. The sequence number of cecal microorganisms was sufficient and can be used for data analysis.

Related studies have proved that Thick-walled bacteria are related to the ability to obtain energy (48), and the ratio of Thick-walled bacteria to *Bacteroidetes* is significant for maintaining the homeostasis of the internal environment and the utilization of nutrients (49). Increasing *Firmicutes* and decreasing *Bacteroidetes* in cecum contents can improve nutrient absorption efficiency. In this study, the dominant bacteria at the phylum level in each experimental group were *Firmicutes, Bacteroidetes*, and *Proteobacteria* (including *Escherichia coli, Salmonella, Helicobacter pylori, Vibrio*, and other pathogens). It indicated that adding antimicrobial peptides to the diet could improve the utilization rate of nutrients, consistent with the result that the antimicrobial peptides could improve the growth performance of the yellow feather broiler. Moreover, PICRUSt function prediction analysis showed that adding maggot antimicrobial peptides to feed could improve the biological processes of intestinal energy production and transformation, amino acid transport and metabolism, and carbohydrate transport and metabolism. This indicated that the addition of maggot antimicrobial peptides could not only improve the protein utilization ability of yellow-feather broiler but also improve the intestinal metabolic biological process.

## Conclusions

Our study showed that adding maggot antimicrobial peptides to the basal diet could increase average daily gain, reduce the feed conversion ratio of yellow-feathered broilers, and improve their growth performance of yellow feathered broilers. Moreover, adding 300 mg/kg of maggot antimicrobial peptide to the basal diet can improve the blood parameters of serum total protein, IgG, IgA, and the thymus and spleen index of yellow-feathered broilers. It indicated that maggot antimicrobial peptide could improve the immune function of yellow-feathered broilers. The results of 16S rDNA sequencing of cecum intestinal flora showed that the maggot antimicrobial peptide was beneficial to the intestinal health of yellow-feathered broilers. Therefore, the appropriate dose for adding antimicrobial peptide is 300 mg/kg.

## Data availability statement

The raw data supporting the conclusions of this article will be made available by the authors, without undue reservation.

## Ethics statement

The animal study was reviewed and approved by Shihezi University.

## Author contributions

Conceptualization: HS and SG. Methodology, investigation, and writing—original draft preparation: SG. Software: QZ. Validation: HS, SG, and QZ. Formal analysis: CL. Writing—review and editing, project administration, and data curation: HS and JW. All authors have read and agreed to the published version of the manuscript.
